# Adaptive threshold segmentation of pituitary adenomas from FDG PET images for radiosurgery

**DOI:** 10.1120/jacmp.v15i6.4952

**Published:** 2014-11-08

**Authors:** Hannah M. Thomas T, Devakumar Devadhas, Danie K. Heck, Ari G. Chacko, Grace Rebekah, Regi Oommen, E. James J. Samuel

**Affiliations:** ^1^ Photonics, Nuclear and Medical Physics Division School of Advanced Sciences, VIT University Vellore India; ^2^ Department of Nuclear Medicine Christian Medical College Vellore India; ^3^ Department of Neurosurgery Christian Medical College Vellore India; ^4^ Department of Biostatistics Christian Medical College Vellore India

**Keywords:** pituitary adenoma, adaptive threshold, segmentation, positron emission tomography

## Abstract

In this study we have attempted to optimize a PET based adaptive threshold segmentation method for delineating small tumors, particularly in a background of high tracer activity. The metabolic nature of pituitary adenomas and the constraints of MRI imaging in the postoperative setting to delineate these tumors during radiosurgical procedures motivated us to develop this method. Phantom experiments were done to establish a relationship between the threshold required for segmenting the PET images and the target size and the activity concentration within the target in relation to its background. The threshold was developed from multiple linear regression of the experimental data optimized for tumor sizes less than 4 cm^3^. We validated our method against the phantom target volumes with measured target to background ratios ranging from 1.6 to 14.58. The method was tested on ten retrospective patients with residual growth hormone‐secreting pituitary adenomas that underwent radiosurgery and compared against the volumes delineated by manual method. The predicted volumes against the true volume of the phantom inserts gave a correlation coefficient of 99% (p<0.01). In the ten retrospective patients, the automatically segmented tumor volumes against volumes manually delineated by the clinicians had a correlation of 94% (p<0.01). This adaptive threshold segmentation showed promising results in delineating tumor volumes in pituitary adenomas planned for stereotactic radiosurgery, particularly in the postoperative setting where MR and CT images may be associated with artifacts, provided optimization experiment is carried out.

PACS number: 87.57.nm, 87.57.uk

## I. INTRODUCTION

Although PET imaging is now actively used for diagnosis, staging, and tumor response evaluation, its role in treatment planning for delineation of radiotherapy target volumes is still limited clinically. One of the challenging issues limiting its use is the poor resolution of PET images which make distinguishing the edge of the tumor a difficult task. The other issue is the absence of an easy‐to‐implement automated delineation methods to delineate the tumor. The methods can be classified as manual, semiautomatic, and fully automatic segmentation techniques. Manual segmentation is observer‐dependant, as the delineation of the target could be a subjective interpretation of image by the clinician. Inter‐ and intraobserver variability in the target delineating has been reported and variability also was largely affected by the experience of the clinician. This process is also quite slow and has poor reproducibility.^(1)^ PET images are susceptible to variations in window level settings,^2^, ^3^, ^4^ and changing the intensity of the images or the color scale dramatically changes the perception of the volume of the target.^(5)^ In spite of these problems with manual segmentation, it still remains the most common method of delineation, probably because it is very simple and does not need any optimization.

During recent years, our institution has been using FDG PET in imaging pituitary adenomas in the postoperative settings. Pituitary adenomas comprise about 10% to 15% of all diagnosed intracranial tumors^(6)^ and frequently require radiotherapy after transsphenoidal surgery to achieve a cure in functional adenomas or to arrest growth in residual nonfunctional adenomas. While magnetic resonance imaging (MRI) is the standard imaging modality used for these tumors, delineating tumors in a postsurgical setting is difficult due to artifacts from old blood, fat used for packing the sella, and haemostatic material.^(6)^ The metabolic nature of pituitary tumors makes them good candidates for functional imaging using FDG PET^7^, ^8^ and may be useful during radiosurgical procedures for pituitary adenomas given the constraints of MRI in the postoperative setting. In stereotactic radiosurgery (SRS), a single radiation beam or a limited number of high‐dose beams are delivered to the small, precisely defined target avoiding critical structures, such as the optic chiasm and optic nerves that lie within a few millimeters of the target. Although there is a sharp dose falloff beyond the target, the need for precise definition of the target cannot be overemphasized.

As a result, we tried to automate the target delineation of the pituitary adenomas as the functional uptake of interest and avoid the inclusion of non tumor‐specific activity uptake. Many automatic segmentation methods have been suggested in literature over the last decade, but only fixed threshold‐based segmentation algorithms are available in commercial treatment planning systems (e.g., Eclipse from Varian). A number of clinical papers^9^, ^10^, ^11^ still report work done using fixed threshold methods like absolute value of SUV (e.g., SUV=2.5,3,5) or percentage of the maximum intensity (40% or 50%), though it has largely been proven to be an oversimplification.^12^, ^13^, ^14^, ^15^ Adaptive threshold methods based on calibrated curves giving the threshold as a function of source‐to‐background ratio are superior to fixed threshold‐based segmentation^16^, ^17^, ^18^, ^19^ and are intuitively simpler to implement, especially in a setting of limited medical physics support. The purpose of our work is to add to the existing knowledge and report our findings in obtaining an optimal threshold required to segment small tumors. Our adaptation has been using multiple regression method; we also tried to simplify the method described by Jentzen et al.^(20)^ and have compared the results. If the subjectivity in drawing the background region in this algorithm be further reduced, both multiple regression algorithm and the modified Jenzten method can be implemented in the TPS for segmentation that has fixed threshold option by calculating the required threshold using a spreadsheet calculator.

## II. MATERIALS AND METHODS

### A. Data acquisition

A Siemens PET CT scanner (Biograph 6 TruePoint HD; Siemens AG, Munich, Germany) was used for acquiring and reconstructing the PET/CT images. The field of view of the scanner was 162 mm. The acquisition and reconstruction protocols routinely used for clinical studies were used for the phantom study. The CT images were first acquired at 90 mAs, 80 kV (p), with 3 mm collimation and pitch of 0.5. The slice thickness was 3 mm per slice. The PET images were acquired in three‐dimensional mode with a ring difference of 27 and span 11. Two bed positions of 15 min each were used for imaging the phantom since the entire phantom could not be covered in a single bed position.

The sinogram data were decay, dead time, and random corrected. Image reconstruction was performed using the ordered subsets expectation maximization algorithm. During the reconstruction, corrections for point spread function (TrueX), attenuation, and scatter were performed. The CT images were used for the attenuation correction. The reconstruction was performed with three iterations with 21 subsets and the zoom factor was adjusted to 2. The post processing filter used was a three‐dimensional Gaussian with 2 mm full width at half maximum (FWHM). The resultant PET image size was 336×336 pixels each with a slice thickness of 3 mm and 1.02×1.02mm in the transaxial plane. The same acquisition and reconstruction parameters were used for the patient imaging, except the imaging time was 10 min in a single bed position.

### B. Phantom description

**Figure 1:**
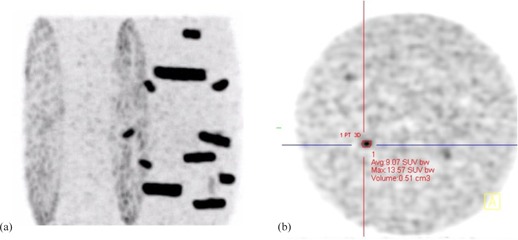
Maximum intensity projection of phantom (a); axial view (b) of the PET image.

The phantom study was done to establish a relationship between the threshold required for segmenting the PET images and both the size of the target and the activity concentration within the tumor with respect to its background. The volumes addressed in this experiment were in tune to the range 0.5 to 4 cm^3^ to account for small tumors. Since the Jaszczak phantom (Data Spectrum Corp., Durham, NC) did not have inserts to hold very small volumes, refillable containers of volume 0.5, 1, 2, and 4 cm^3^ were fixed to the acrylic rods on the inner aspect of the lid of the phantom using rubber bands. To account for the effect of target‐to‐background (T/B) ratio on the threshold, all containers were filled with activity using a pipette to give a target‐to‐background ratio of 3.5, 5, and 13 in three separate acquisition sessions. Since it was difficult to reproduce the exact T/B ratio in consequent phantom preparation, we used three containers for each volume with the same T/B ratio for each acquisition session. This helped to obtain more readings in a single acquisition. The phantom was placed on its side in the gantry. So the small containers were horizontal in orientation, and transverse slices of the containers were obtained after the reconstruction (Fig. 1(a)).

### C. Baseline threshold calculation

Reconstructed images were displayed and analyzed in the oncology software engine Syngo MMWP VE 36A (Siemens AG) workstation. The display was in standard uptake values (SUV). The window and level settings were adjusted individually for each of the inserts in question to view the target at its maximum intensity. The ratio of maximum tracer activity in the target (T) and the mean activity in background (B) surrounding the target was measured from the images. The targets were the inserts containing the radioactivity. An ellipsoid tool was used as a bounding box to limit the volume of interest (VOI) for segmentation.

The ellipsoid was first drawn on a single axial slice (Fig. 1(b)) and viewed simultaneously on the coronal and sagittal views to verify the extent. The size of the ellipsoid was adjusted such that the entire container was within the ellipsoid. An average background (B) was estimated by placing multiple small ellipsoidal volumes in close proximity around the target. The optimal threshold for arriving at the true insert volume was found by manually varying the threshold until the true volume was reached. This threshold was taken as the baseline and would be henceforth referred to as so. The T/B ratio and baseline threshold were calculated for all the 12 containers in each of the three different ratios considered (3.5, 5, 13). This baseline threshold was used to arrive at the threshold from the predictor equations, as described in later sections.

### D. Threshold predictor equations

#### D.1 Method 1: Multiple linear regression method

We used multiple linear regression to arrive at a predictor equation for threshold based segmentation of the target that took into account the size of the target, the concentration of tracer accumulated inside the target (T), and the background activity (B) near the target. The predicted entity being the threshold (baseline threshold) was taken as the dependent variable. The variables (1‐measured B/T) and (1/actual volume) that affect the threshold were taken as the independent variables. The beta coefficients and p‐values were calculated for both the independent variables. $R^{2}$ was used to report the proportion of variance in the values of the predicted threshold due to the independent variables. SPSS Version 16.0 was used for analysis.

The transformation (1‐B/T), as suggested by Brambilla et al.,^(21)^ and the inverse of size of the target expressed in cm^3^ showed a linear relationship with the baseline threshold. So, a linear model with these two variables of the form T=k+a∗(1−B/T)+b∗(1/size) was assumed as the predictor equation for threshold, where *k, a, b* are the regression coefficients. The values for the regression coefficients were obtained by fitting the experimental data. The statistical details of the regression analysis are mentioned in the Results section below. The predictor equation developed was:

(1) T(%)=94.933−73.938*(1−measured B/T)+2.739*(1/Actual volume)

When this equation was applied in clinical situations, the T/B ratio could be obtained from the image as described earlier, but there is no a priori knowledge of the target (the inserts) and its volume. So we used a simple linear regression method to get an equation that predicted a threshold solely dependent on the B/T ratio of the tracer activity measured from the PET image. On fitting the curve with linear regression we obtained Eq. (2) that set the threshold to obtain a volume:

(2) T(%)=−77.89*(1−measured B/T)+100.4

The volume resulting from this threshold was referred to as the initial volume estimate.

The predictor Eq. (1) was modified as follows to include this initial volume parameter instead of actual target volume:

(3) T(%)=94.933−73.938*(1−measured B/T)+2.739*(1/Initial volume estimate)

All volumes resulting from Eqs. (1), (2), (3) were measured in cubic centimeters. Equation (3) was used to validate the phantom and retrospective patient data.

#### D.2 Method 2: Modified Jentzen method

Jentzen et al.^(20)^ suggested an iterative threshold method where the threshold predictor was a simple linear equation of the form T=m/V+T1, where *m* is the slope, *V* is the volume of target, and *T1* is the intercept. The first term (m/V) on the right side of the equation accounts for size of the tumor, and the second term (T1) accounts for target to background ratio of the tumor. The intercept T1 was a linear‐fitted equation obtained from separate parameterization curves for different T/B ratios considered. We implemented this method with a few modifications using the data generated from our phantom experiments. A detailed description for obtaining the intercepts and the equation for estimating the initial volume (Eq. (3A) is given in the Appendix.

To arrive at the final equation accounting for both volume and concentration, we differed from Jentzen et al.^(20)^ since we considered the Eq. (3A) representing the highest T/B ratio 13, while they used the equation corresponding closest to the T/B ratio in question for each target. Equation (3A) was taken as the base equation because this equation would give the lowest possible threshold when the activity concentration is the highest (T/B ratio 13) and, when other T/B values are used, the equation would give a higher threshold value accordingly.

We replaced the intercept part of Eq. (3A) with the equation (4A) which led to Eq. (4):

(4) T(%)=2.666*(1/Actual volume)+65.278*(Actual B/T)+21.949

Since the actual B/T ratio and the actual volume are not known when used for tumor delineation in patients, the Eq. (4) was modified as

(5) T(%)=2.666*(1/Initial volume estimate)+65.278*(measured B/T)+21.949

where measured *B/T* is the ratio of the mean SUV value in the background region of interest surrounding the tumor and SUVmax in the tumor region, both measured from the image. The initial volume estimate is calculated from Eq. (4A).

### E. Validation on phantom

Both the threshold predictor Eqs. (3), (5) were first validated against a separate set of phantom data. In Method 1, the B/T value from the image for each radioactivity container was measured and used in Eq. (2) to get the threshold for initial volume estimate. The threshold for the initial volume estimate was used for the segmentation of radioactivity inside the container and the volume of the segmented region was found (initial volume estimate) that was substituted in Eq. (3). The final threshold was obtained from Eq. (3) for the multiple linear regression method. For the modified Jentzen method (Method 2), the initial volume estimate was calculated from Eq. (4A), but the actual B/T was replaced by B/T measured from the image. The final threshold was estimated from Eq. (5). The residual volume was calculated as the difference between the actual volume and the estimated volume for each method (Table 1).

**Table 1:** Tumor volume comparison for retrospective patients who underwent radiosurgery for pituitary tumor.

	*Multiple Regression Method*					*Modified Jentzen Method*					
*Patient #*	*Initial thres*.	*Initial vol (cc)*	*Final thres*.	*Calc. vol (cc)*	*Volume diff. (cc)*	*Initial thres*.	*Initial vol (cc)*	*Final thres*.	*Calc. vol (cc)*	*Volume Diff. (cc)*	*Manual Delin. Vol (cc)*
1	69	0.38	72	0.28	0.14	61	0.55	66	0.3	0.12	0.42
2	64	0.74	64	0.74	−0.21	57	0.86	60	0.8	−0.27	0.53
3	68	0.44	70	0.37	0.11	60	0.75	63	0.6	−0.12	0.48
4	73	0.49	75	0.43	−0.16	65	0.72	68	0.62	−0.35	0.27
5	75	0.28	81	0.22	0.12	66	0.42	72	0.32	0.02	0.34
6	66	1.68	64	0.77	−0.28	59	1.86	60	0.85	−0.36	0.49
7	56	1.02	56	0.32	0.02	50	1.46	52	1.3	−0.96	0.34
8	41	4.4	39	4.74	−1.31	37	5.12	38	5	−1.57	3.43
9	46	1.39	46	1.39	−0.36	42	2.02	43	1.95	−0.92	1.03
10	45	1.83	44	1.86	−0.95	41	1.9	42	1.88	−0.97	0.91

Volume difference = Manual delineated volume ‐ Calculated volume.

The performance evaluation statistical tests were used to determine the error in the volumes predicted, the consistency in predicting the threshold value for segmentation within the optimized range of volumes and the overall comparison of the methods. The mean square error was obtained for the volumes calculated for both methods against the true volume. The Wilcoxon signed‐rank test was done for each T/B ratio to assess the mean rank difference in the volumes calculated using the two methods. This test was also run to compare the methods to check if there was a consistent under‐ or overestimation of the volume calculated. The intraclass correlation was conducted to ascertain the consistency of the volumes calculated by both the methods against the true volume of the radioactive inserts in the phantom for each T/B ratio. To evaluate the performance of the methods compared with each other, the Bland Altman plot was done, which helped to ascertain if there were any anomalies in the volumes measured.

### F. Validation on patient

**Figure 2:**
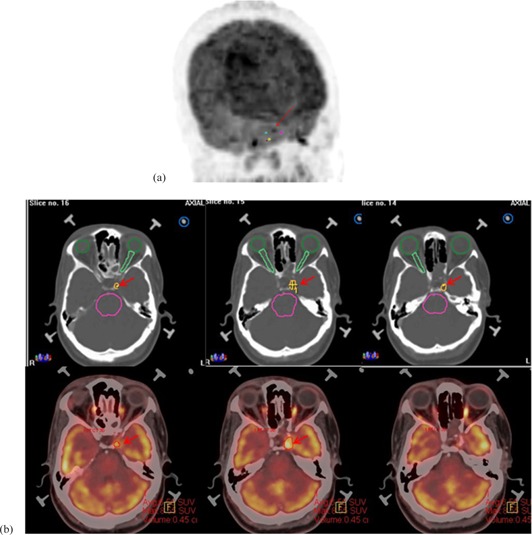
MIP image (a) depicting placement of background around tumor in question. Segmented pituitary adenoma (b): Row 1 – manually delineated volume as segmented by the neurosurgeon; Row 2 – automatic segmentation volume using multiple regression‐based threshold method.

The PET images of ten patients who underwent radiosurgery for pituitary tumor were analyzed retrospectively by the nuclear medicine physicists who were blinded to the manual delineation volume and the tumor segmentation was done using Eqs. (3), (5). An ellipsoid was drawn as the bounding box for the tumor of interest and the SUVmax (T) was obtained. Multiple ellipsoidal volumes of interest were placed around the pituitary adenoma target for background estimation (Fig. 2(a)) and the number of ellipsoids varied from two to four, depending on the intensity variation of the structures around the tumor. When the target was closer to the temporal lobe, maximum of four ellipsoids were used, with at least two close to the temporal lobe. An average background (B) was estimated from SUVmean in each ellipsoid. The T/B value was calculated from the ratio of SUVmax of the target and the background. The threshold values obtained from Eqs. (2), (4A) were used for initial segmentation of tumor and the initial volume estimate was obtained. The T/B ratio and the initial volume estimate were substituted in the predictor Eqs. (3), (5) to obtain the threshold value for automatically segmenting the tumor using both the threshold predictor methods and are tabulated in Table 2.

**Table 2:** Phantom validation data using threshold predictor methods: multiple regression method and Modified Jentzen method.

*Phantom Measurements*				*Method 1: Multiple Regression Method*					*Method 2: Modified Jentzen Method*				
*Actual T/B ratio*	*Measured T/B*	*Actual vol. (cc)*	*Baseline thres*.	*Initial Thres*.	*Initial vol. (cc)*	*Final thres*.	*Calc. vol (cc)*	*Error*	*Initial Thres*.	*Initial vol. (cc)*	*Final thres*.	*Calc. vol (cc)*	*Error*
13	9.89	0.5	34	30	0.57	33	0.5	0	29	0.66	33	0.5	0
	11.87	0.5	30	29	0.51	33	0.43	0.07	27	0.59	32	0.45	0.05
	11.13	0.5	34	30	0.58	32	0.51	−0.01	28	0.66	32	0.51	−0.01
	11.53	1	31	29	1.06	30	1.02	−0.02	28	1.05	30	1.02	−0.02
	13.31	1	27	28	0.95	29	0.9	0.1	27	1.02	29	0.9	0.1
	10.57	1	30	30	1.01	31	0.96	0.04	28	1.02	31	0.96	0.04
	13.13	2	28	29	1.92	28	2.02	−0.02	27	2.08	28	2.02	−0.02
	11.06	2	32	30	2.23	29	2.33	−0.33	28	2.44	29	2.33	−0.33
	13.06	2	32	29	2.15	28	2.25	−0.25	27	2.38	28	2.25	−0.25
	14.22	4	27	28	3.84	27	3.95	0.05	27	4	27	3.95	0.05
	14.58	4	27	28	3.96	27	4.08	−0.08	26	4.17	27	4.08	−0.08
	14.15	4	26	28	3.77	27	3.88	0.12	27	3.85	27	3.88	0.12
5	3.57	0.5	51	44	0.67	46	0.62	−0.12	40	0.82	43	0.68	−0.18
	3.08	0.5	49	48	0.55	50	0.48	0.02	43	0.74	47	0.59	−0.09
	2.69	0.5	57	52	0.72	52	0.72	−0.22	46	1.22	48	0.97	−0.47
	4.39	1	40	40	1.01	41	0.96	0.04	37	1.2	39	1.1	−0.1
	5.15	1	33	38	0.77	39	0.73	0.27	35	0.88	38	0.77	0.23
	3.87	1	42	43	1.04	43	1.04	−0.04	39	1.27	41	1.16	−0.16
	3.85	2	49	43	2.72	41	3.04	−1.04	39	3.23	40	3.19	−1.19
	4.41	2	40	40	2.02	39	2.11	−0.11	37	2.5	38	2.21	−0.21
	5.15	2	34	38	1.73	37	1.8	0.2	35	1.92	36	1.89	0.11
	4.86	4	40	39	4.09	37	4.44	−0.44	35	5	36	4.61	−0.61
	5.32	4	34	37	3.5	36	3.68	0.32	34	4.17	35	3.87	0.13
	5.51	4	33	37	3.46	35	3.77	0.23	34	3.85	34	3.89	0.11
3.5	1.65	0.5	71	70	0.41	72	0.34	0.16	62	1	64	0.7	−0.2
	1.59	0.5	72	72	0.5	73	0.46	0.04	63	1.19	65	0.83	−0.33
	1.8	0.5	69	66	0.7	66	0.7	−0.2	58	1.54	60	1.09	−0.59
	2.67	1	46	52	0.68	53	0.85	0.15	46	1.05	49	0.85	0.15
	2.29	1	56	57	0.99	56	1.04	−0.04	50	1.47	52	1.27	−0.27
	2.3	1	55	56	0.84	56	0.84	0.16	50	1.39	52	1.08	−0.08
	2.84	2	51	50	2.14	48	2.36	−0.36	45	2.94	46	2.59	−0.59
	2.61	2	52	52	1.89	51	1.97	0.03	47	2.78	48	2.33	−0.33
	2.49	2	53	54	1.84	52	2.04	−0.04	48	2.56	49	2.39	−0.39
	3.14	4	42	47	3.14	45	3.39	0.61	43	3.85	43	3.72	0.28
	3.26	4	43	46	3.41	44	3.74	0.26	42	4.35	43	3.93	0.07
	3.21	4	44	47	3.45	45	3.73	0.27	42	4.35	43	4.13	−0.13

Error = (Actual Volume‐Calculated Volume).

For the ten retrospective patient data, correlation test and intraclass correlation coefficients tests were done to compare the volumes calculated by the two predictor Eqs. (3), (5) with the manual delineation volume.

## III. RESULTS

### A. Baseline threshold calculation

**Figure 3:**
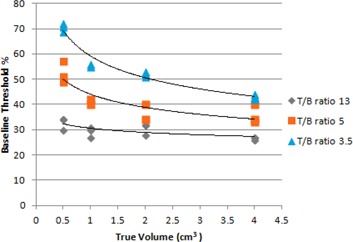
Change in threshold with effect to change in volume with different T/B ratio.

From the observations from the phantom experiments it is seen that, though a fixed concentration of 13, 5, and 3.5 were used (Table 2), the measured T/B ratios varied within +3 of the actual value, which could be due to spillover and spill in related to the partial volume effect. Figure 3 shows that there is a relationship between the actual volume and baseline threshold for varying concentration ratios. As the volume increased, the baseline threshold decreased. When the concentration ratio between the target and background is low, the resolution of the image decreased, increasing the image blur. This led to a larger threshold being required to the segment smaller volumes than what is visually seen. This is similar to the relationship observed and reported by others.^20^, ^21^

### B. Noise properties in phantom and patient data

The measured tracer concentration depicted as SUVmax in the inserts was between 7.1 and 14.6 for T/B ratio of 3.5, between 11.6 and 20 for T/B ratio 5, and between 37.5 and 55 for T/B ratio 13. The mean SUV values in the background for the three experiments ranged between 3.5 and 4.

The measured tracer concentration in the pituitary adenoma targets represented by the SUVmax values ranged from 3.4 to 12.6. The background concentrations measured as SUVmean were between 1.2 and 5. Since the phantom experiments were designed and optimized for the SUVmax and SUVmean values seen in pituitary adenomas and its surrounding structures, respectively, the noise properties in the phantom are a true representation of the routine clinically observed noise properties. Hence, the adaptive threshold equation obtained from the phantom data would give a realistic threshold value for segmentation of pituitary adenomas in clinical situation. So, the measured tracer concentration ratios in the patient data varied between 1.8 and 4.25, which was within the optimized range of measured T/B ratios in the phantom.

### C. Multiple regression model

**Figure 4:**
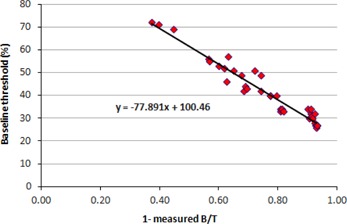
Plot of baseline threshold vs. 1/volume.

**Figure 5:**
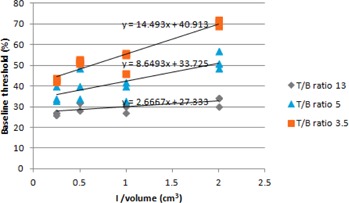
Plot of (1‐measured B/T) vs. baseline threshold.

The $R^{2}$ value is 0.96 $\left(\text{adj}\;R^{2}=0.958\right)$ for the multiple linear regression model, which shows that both the independent variables T/B ratio and target size showed good correlation with the baseline threshold. Both these predictor variables are statistically significant whose variance can be accounted for primarily by the T/B ratio (β=−0.92) and secondarily by the target size (β=0.15). The positive beta value for the variable (1/actual volume) shows that there is linear relation with the threshold (Fig. 4). Similarly, the negative beta value for (1‐measured B/T) suggests that it has an inverse relationship with the threshold (Fig. 5). So the slopes shown in Figs. 4, 5 are from simple linear regression, while the beta values are obtained from multiple linear regression.

### D. Phantom

The validation of the volume of the inserts in the phantom experiment measured by using the threshold predictor equations for both the multiple linear regressions and the modified Jentzen method against the true volume of the inserts are shown in two independent columns in Table 2. The three concentration ratios used have been classified separately as the actual T/B ratio. The initial estimate of the volume obtained using the corresponding initial threshold equations for each method (Eqs. (2), (5)) have also been shown under each method separately. The error in predicting the true volume is also shown under each method. It was seen that Methods 1 and 2 have approximately the same error in volume estimated for T/B ratio of 13. For both methods the volume was under‐ or overestimated with decrease in the T/B ratio.

Mean square error was obtained for the volumes calculated from both methods for the three T/B ratios: T/B ratio 13 did not show any difference (mean=0.177) in both methods, while T/B ratio 3.5 showed a mean square error of 0.06 and 0.1, and T/B ratio 5 had mean square errors as 0.13 and 0.18 for Methods 1 and 2, respectively.

When the Wilcoxon signed‐rank test checked the performance of both methods independently against the actual volume of the inserts, the p‐value for T/B ratio 13 was 0.317. This suggests that the predicted values were very close to the actual value for T/B ratio 13. Significant difference was found in the volumes predicted for T/B ratios 5 and 3.5 in both methods, with the p‐values being 0.02 and 0.03, respectively. When the methods were compared with each other, the volumes were overestimated by the modified Jentzen method at the lowest concentration ratio (T/B ratio 3.5).

The intraclass correlation showed excellent correlation of measured and true volume for both methods at T/B ratio 13 with ICC=1.0. T/B ratios 3.5 and 5 gave ICC=0.983 and ICC=0.977 in Method 1 and ICC=0.979 and ICC=0.961 for Method 2, respectively. Intraclass correlation between the methods also suggested that the multiple linear regression method was consistent in predicting a threshold value close to the baseline threshold, even at lower concentration ratios. We can conclude from these tests that the multiple linear regression method showed a better overall performance.

The Bland Altman plot showed that all the predicted volumes using both methods lie within the mean ±2SD.

### E. Validation on patient data

The volumes of the ten retrospective patients calculated using the two segmentation methods were within 0.22 to 4.74 cm^3^ for multiple regression method and between 0.3 and 5 cm^3^ for the modified Jentzen method. The manual delineation tumor volumes treated ranged from 0.27 to 3.43 cm^3^. The calculated volumes from the two methods correlated with the manual delineation volumes with similar correlations of 94% and p<0.01. The intraclass correlation coefficients for volumes from the multiple regression and the modified Jenzten methods were in close agreement with the manual delineation volume, with correlation coefficients being 0.938 and 0.934, respectively. Overestimation of volume was seen in both multiple regressions and modified Jentzen method for patients who had tumors which extended beyond the sella region. Figure 2(b) depicts the tumor volumes delineated both manually and using adaptive threshold method for a tumor confined to the left cavernous sinus. To avoid interobserver variability, the manual delineation was done by the same neurosurgeon in all patients considered in this study.

## IV. DISCUSSION

The process of distinguishing the tumor edge from adjacent structures or background is known as segmentation. Identifying the tumor background interface is straightforward when there is relatively high FDG uptake in the tumor as compared to the background; but when the uptake is similar, segmentation becomes difficult particularly when performed manually. The challenge at hand was to segment the pituitary adenomas that are both small and also set against a high‐intensity background. Intuitively, since the images show a marked difference between the normal and abnormal glycolytic functions, thresholding was one of the easier methods to adopt and implement,^22^, ^23^, ^24^, ^25^ provided it is optimized for the specific scanner. Since we were delineating small tumors, fixed threshold was not an ideal option, given its own limitations.^(26)^ Since this data‐driven adaptive threshold method is based on the simple principle that the required threshold for segmentation is inversely proportional to T/B, it is easily understood by an average clinical user than achieving an intuitive understanding of the mathematical models for clinical implementation. So the experiments were designed for the range of tumor volumes we normally treat in radiosurgery and for the noise parameters clinically seen. Initially, the idea was to implement a method based on literature available for delineating small tumors. But selecting an ideal method was a difficultly in itself.

Firstly, the parameters that contributed largely towards the optimal selection of an adaptive threshold to delineate the tumors in question varied considerably. The theoretical model that van Dalen et al.^(27)^ derived obtained a background subtracted relative threshold method which is independent of the source to‐background ratio but dependent on the sphere size, and suggested a threshold level >50% for spheres of diameter <12mm. Biehl et al.^(16)^ found that appropriate threshold for PET‐GTV delineation is highly dependent on tumor size, and arrived at optimum threshold ranges of about 42%±2% for tumors measuring less than 3 cm. Black et al.^(17)^ reported a method where the threshold was dependent only on the SUV mean of the target and not related to the background activity or the tumor volume. Erdi et al.^(28)^ reported that fixed thresholds could be obtained from the SBR curves to arrive at the threshold. It was Jentzen et al.^(20)^ who formulated that optimal threshold to segment lesions were dependent on both SBR and lesion size. In our own experiments we found it to be relevant to the tumors in question. Brambilla et al.^(21)^ also found similar relation and concluded that parameters like injected activity and scan durations had a negligible role in predicting an optimal threshold.^(18)^ Based on suggestions from their work for the clinical implementation, we used the activity ratio of target to its background as measured from the images and not the actual T/B as used in the phantom.

We found that the multivariate approach by Brambilla and colleagues is a better formulation when we have more than one predictor variable contributing to the dependent variable which, in our case, was the optimum threshold. This method is better than the method followed by Jentzen et al.,^(20)^ where they used simple linear regression to combine the effects of the two dependent variables using linear regression equations arrived independently from the calibrated curves. This approach does not fully take into consideration the weighting parameters of the independent variables to the extent of their contribution.

As the equation requires the volume of the target to compute the threshold, which in clinical situations is the unknown variable, it was difficult to use the formulation of the equation suggested by Brambilla et al.^(21)^ in its original form. Erdi et al.^(28)^ obtained the approximate lesion size from the CT and T/B ratio from PET to obtain the appropriate optimum threshold which was applied to the PET images to obtain lesion activity and a final estimate of the lesion volume. But this method cannot be used in lesions only seen on the PET. Jentzen et al.^(20)^ had attempted to estimate the PET volumes by using a fixed threshold from the calibrated curves closest to the measured SBR for iteratively calculating the volume. It was done by using a fixed threshold which is the lowest threshold value from the corresponding curve. Instead we developed a simple linear regression equation involving only the T/B ratio measured from the image to roughly estimate the volume of the questionable lesion.

Apart from the results of the predictor equations, we would like to discuss some salient observations made during the study. When considering the tumor sizes ranging from 0.5 to 4 mm, the model fitting was good $\left(R^{2}=0.96\right)$ with the most relevant contributor to the threshold being the T/B ratio (β=−0.92). The tumor size contributes less to the threshold prediction when compared to T/B ratio (β=0.15), but still significantly. This trend is similar to that reported by Brambilla et al.^(21)^ and Drever et al.^(29)^

It was observed that, for the smallest volume (0.5 cm^3^) at the lowest concentration ratio (T/B=3.5), the SUV gradient gave a wider Gaussian‐like curve and hence higher full width at half maximum (FWHM) when a profile is plotted across the container region. The low signal‐to‐noise ratio (SNR) increases the uncertainty in observed SUV value, which, in turn, increases the width of the Gaussian curve. Thus, the image of the container or tumor appears bigger than its actual size.^(26)^ To obtain the true volume of the container or tumor during the segmentation process, a smaller volume compared to the visible volume of the image needs to be marked, which is done by using a higher threshold. In our study, the highest threshold used was 72% for the container with the lowest concentration. Increasing the concentration of the tracer improved the SNR and reduced the FWHM. For the highest concentration container, the true volume may be obtained by using a lower threshold, which is 30% in this study. The poor resolution also led to an underestimation of the maximum activity for all volumes considered. With decreasing volumes there was a substantial increase in the underestimation of the threshold leading to an increase in the predicted volume of the target, which is largely due to partial volume effect. If the resolution of the reconstructed image is improved by increasing the number of iterations or post filter cutoff and partial volume correction or deblurring, then the threshold required will be less.

There are a few limitations of our study that include the obvious, that the adaptive threshold segmentation method developed is specific for the PET scanner and the reconstruction parameters mentioned in the Materials and Methods section. We did not use spherical inserts in the Jaszczak phantom as commonly seen in literature, due to its nonavailability. Instead, small vials of known volumes were used, ensuring that no air was in the container (Fig. 1(a)). Since pituitary tumors are not resected en masse as in the case of other solid tumors, it is not possible to estimate the true volume of the resected specimen. Therefore, validation based on phantom experiments seemed to be the best tool possible. For future studies, we intend to validate the method in totally resectable small tumors.

The primary limitation of the method is the dependence on the selection of the background. Schaefer et al.^(30)^ suggested drawing regions of interest (ROI) in every plane between the spheres at a distance of at least 5 mm from the target, and the mean SUV of all these ROIs was used as background value (BG). But from our experiments we found that determining the background in the immediate vicinity of the target under consideration yields better delineation results. Instead of using ROIs in various planes, we used ellipsoidal volumes of interest in close proximity to the target. When the background heterogeneity was high, the number of ellipsoids was increased to four from two which was used when the pituitary mass was well confined within the sella. Our future work includes methods to reduce the subjectivity in drawing the background.

The method to estimate the initial volume is simple compared with other more tedious procedures.^(20)^ The volumes segmented by this method in patient data showed a good correlation (0.94) with the manually delineated volumes. A detailed study may be required with more realistic physical phantom and simulations with a realistic digital phantom. As with all automatic segmentation techniques, there should be necessary operator intervention to verify the regions delineated.

While we observed in our study that the volume of tumor from adaptive threshold segmentation and manual segmentation is similar, the edges obtained from the two segmentation methods may be different. Future studies with improved dice similarity, coefficient estimate may be required to verify the matching of the edges of the tumor obtained from different segmentation methods. Though this method makes it possible to delineate small tumors with ease rather than using manual segmentation, some uncertainty may exist because of the reasons mentioned above. Future efforts will test this method on other PET systems. The method has been described for easy reproduction of the results in any clinical setting, provided the imaging parameters and imaging system considered are replicated.

## V. CONCLUSIONS

The proposed adaptive threshold segmentation method is optimized for small tumors less than 4 cm^3^. Application to the clinical situation is relatively simple as it involves only two equations: one for the threshold calculation for initial volume estimate developed using simple linear regression, and a second equation for the threshold calculation of the final volume developed by multiple linear regressions of the experimental data using the phantom. It was compared with a modified version of the threshold‐based segmentation method suggested by Jentzen et al.^(20)^ and was seen to perform equally well. Implementation of our method on retrospective patient data from pituitary adenomas treated with stereotactic radiosurgery resulted in calculated segmented tumor volumes correlating well with the manually segmented tumor volume. PET‐based adaptive threshold segmentation method developed by multiple linear regression of experimental data is a promising method for delineation of small tumor volumes — as in our case pituitary adenomas planned for stereotactic radiosurgery — particularly in the postoperative setting where MR and CT images may be associated with artifacts.

## ACKNOWLEDGMENTS

The authors would like to thank Dr. Prema Muthuswamy for helping with parts of the statistical analysis, and Mr. Timothy Peace and Mr. Sivasakthi for their help with the clinical data. HM Thomas acknowledges CSIR, India for funding the senior research fellowship. The authors wish to acknowledge the anonymous reviewers for their detailed and helpful comments to the manuscript.

## APPENDICES

### Appendix A: The method for estimating the initial volume in the modified Jentzen method

The method is described in detail here. The equation has been used for calculating the threshold for segmenting the final target volume.

For each of the three T/B ratios considered in our phantom experiment, a graph was plotted with the inverse of the volumes of the inserts against their corresponding baseline threshold (see Fig. 4). A linear fit equation was found for each of the curves as given below:

(1A) for T/B ratio 3.5 y=14.493 x+40.913

(2A) for T/B ratio 5    y=8.649 x+33.725

(3A) for T/B ratio 13 y=2.667 x+27.333

where *x* is inverse of the actual volume.

**Figure A1:**
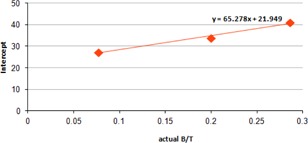
Plot of intercept vs. actual B/T.

We plotted a second graph (Fig. A1) with each actual B/T against the corresponding intercepts obtained from the Eqs. (1), (2), (3A). We fitted a linear curve and obtained the following equation which is used for estimating the initial volume:

(4A) T(%)=65.278 x (Actual B/T)+21.949

The intercept in each of the Eqs. (1A), (2A), (3A) represents the lowest possible value of threshold for the corresponding value of T/B and for the highest volume used in the experiment. So Eq. (4A) obtained from intercepts accounts for T/B ratio and gives the lowest possible threshold corresponding to the highest volume used in our experiments. It also suggests that it is not accounting for size of the tumor if the size of the tumor is less than the maximum volume used in our experiments. So Eq. (4A) gives the minimum possible threshold required for any given B/T ratio lying between 3.5 and 13, and another equation is required to account for volume.

## Supporting information

Supplementary Material
