# Supplementary material for: Adaptive threshold segmentation of pituitary adenomas from FDG PET images for radiosurgery
Source: J Appl Clin Med Phys. 2014 Nov 8;15(6):279–94. doi: 10.1120/jacmp.v15i6.4952 (PMC5711116; doi:10.1120/jacmp.v15i6.4952)
Supplement: Supplementary file 1 — Supplementary Material [file ACM2-15-279-s001.doc]

Adaptive threshold segmentation of pituitary adenomas from FDG PET images for radiosurgery

Hannah Mary Thomas T^1^,

^1^Photonics, Nuclear and Medical Physics Division, School of Advanced Sciences, VIT University, Vellore, India.

*hannahrheathomas@gmail.com*

Devakumar Devadhas^2^

^2^Department of Nuclear Medicine, Christian Medical College Vellore, India

[*devakumar@cmcvellore.ac.in*](mailto:devakumar@cmcvellore.ac.in)

Danie Kingslin Heck^2^

^2^Department of Nuclear Medicine, Christian Medical College Vellore, India

*danie_naz@rediffmail.com*

Ari G Chacko^3^,

^3^Department of Neurosurgery, Christian Medical College, Vellore, India

*agchacko@cmcvellore.ac.in*

Grace Rebekah^4^,

^4^Department of Biostatistics, Christian Medical College, Vellore, India

*gracerebekah@gmail.com*

Regi Oommen^2^,

^2^Department of Nuclear Medicine, Christian Medical College Vellore, India

*regi@cmcvellore.ac.in*

James Jebaseelan Samuel E^1^,

^1^Photonics, Nuclear and Medical Physics Division, School of Advanced Sciences, VIT University, Vellore, India

*ejames@vit.ac.in*

Corresponding author Hannah Mary Thomas T

Running title: Adaptive threshold segmentation from PET
